# Association Between Gut Microbiota and Postoperative Delirium—A Scoping Review

**DOI:** 10.3390/nu18081201

**Published:** 2026-04-10

**Authors:** Izabella Prządo, Patrycja Patronik, Łukasz Karaś, Katarzyna Piekarz, Wioletta Mędrzycka-Dąbrowska, Sabina Krupa-Nurcek

**Affiliations:** 1 Faculty of Medicine, Collegium Medicum, University of Rzeszów, 35-310 Rzeszów, Poland; izabellaprzado@op.pl (I.P.); patronikpatrycja@gmail.com (P.P.); ukasz982@gmail.com (Ł.K.); kattpiekarzz@gmail.com (K.P.); 2 Department of Anaesthesiology Nursing & Intensive Care, Faculty of Health Sciences, Medical University of Gdansk, 80-211 Gdansk, Poland; wioletta.medrzycka-dabrowska@gumed.edu.pl; 3 Department of Surgery, Faculty of Medicine, Collegium Medicum, University of Rzeszów, 35-310 Rzeszów, Poland

**Keywords:** gut microbiota, delirium, postoperative delirium, scoping review

## Abstract

**Background/Objectives**: Postoperative delirium (POD) is a common postoperative complication, especially in the elderly, and is associated with a worsening prognosis, prolonged hospitalization and reduced quality of life of patients. A growing body of research indicates that disorders of the composition of the gut microbiota and dysfunction of the gut–brain axis may play a key role in the pathogenesis of POD. **Methods**: The aim of this review was to assess the association between the gut microbiota and the occurrence of POD. This review was carried out in accordance with the JBI and PRISMA-ScR guidelines, searching for publications in six databases and selecting them according to PCC criteria. Finally, seven works were included in the analysis after an independent assessment. **Conclusions**: The available studies indicate that disorders of the gut microbiota and related metabolic and immune changes may significantly increase the risk of POD. It has been shown that certain bacteria and metabolites, such as SCFAs or indoles, can perform both protective and conducive functions for the development of POD. Understanding these mechanisms opens up the prospect of developing new preventive and therapeutic strategies based on the modulation of the gut microbiota.

## 1. Introduction

Postoperative delirium (POD) is an acute, transient disorder of consciousness, attention, and cognitive function that can occur within a few days after surgery [1]. This condition is particularly common in the elderly and patients undergoing extensive orthopedic or cardiac surgery or oncological surgeries. POD is associated with a significant deterioration in prognosis, increased hospitalization time, increased risk of complications and reduced quality of life of patients. Despite intensive research, the pathogenesis of postoperative delirium remains not fully understood, and effective methods for its prevention and treatment are still limited [2]. In recent years, there has been increasing attention paid to the role of the gut microbiota in the regulation of neurological, immune, and metabolic functions. The gut microbiota, which is a complex community of microorganisms that inhabit the human digestive tract, plays a key role in maintaining the body’s homeostasis [3]. Through the production of short-chain fatty acids (SCFAs), modulation of the inflammatory response, impact on the integrity of the intestinal barrier and participation in the metabolism of neuroactive substances, the microbiota participates in the functioning of the gut–brain axis, a system of two-way communication between the intestines and the central nervous system [2,4]. Microbial imbalances, referred to as dysbiosis, can lead to increased intestinal permeability, translocation of bacterial toxins into the bloodstream, activation of microglia, and the development of neuroinflammatory mechanisms that are strongly associated with the pathogenesis of POD. In addition, the microbiota affects the metabolism of tryptophan, serotonin, dopamine and GABA, as well as the production of metabolites such as indole-3-propionic acid (IPA) or trimethylamine N-oxide (TMAO), which can modulate the risk of delirium [2,5]. Perioperative factors such as surgical stress, antibiotic therapy, dietary changes, immobilization and the patient’s age can significantly affect the composition of the gut microbiota. In the context of the growing interest in microbiotic interventions—such as probiotics, prebiotics or dietary modifications—a better understanding of the relationship between the microbiota and POD is needed, which can open up new possibilities for prevention and therapy [4]. Sarcopenia, defined as a progressive loss of muscle mass and function, is associated with significant changes in the composition and activity of the gut microbiota, leading to a decrease in its diversity and metabolic stability. These disorders promote the predominance of bacteria with pro-inflammatory potential, which can exacerbate systemic inflammation and contribute to further degradation of muscle tissue [6]. At the same time, intestinal dysbiosis limits the production of short-chain fatty acids, such as butyrate, which play a key role in regulating muscle metabolism and energy homeostasis. People with sarcopenia also experience more frequent nutritional deficiencies and reduced fiber intake, which further exacerbates adverse changes in the gut ecosystem [7]. A growing body of data points to the bidirectional nature of this relationship: sarcopenia can initiate or exacerbate dysbiosis, and a disturbed microbiota can accelerate the progression of sarcopenia through inflammatory and metabolic mechanisms. In light of current research, modulation of the gut microbiota—through dietary interventions, prebiotics, probiotics, or synbiotics—represents a promising strategy to support the prevention and treatment of sarcopenia, although it requires further, well-designed clinical trials [8].

The gut–brain axis is a complex, bidirectional communication system between the gastrointestinal tract and the central nervous system, including neuronal, hormonal, immune, and metabolic signaling [9]. In recent years, a growing body of evidence indicates that impaired intestinal delirium—particularly in the context of intestinal dysbiosis—may play a key role in the development of POD [10,11,12]. The perioperative period is associated with a number of factors that destabilize the microbiota, such as surgical stress, antibiotic therapy, dietary changes, immobilization or intestinal perfusion disorders [13,14,15]. The result of these processes is a weakening of the integrity of the intestinal barrier, increased permeability and translocation of bacterial toxins, which can initiate a systemic inflammatory response and affect brain function. One of the most important mechanisms connecting the microbiota to the central nervous system (CNS) is the modulation of the immune response. Dysbiosis leads to increased production of lipopolysaccharide (LPS) and other pro-inflammatory mediators, which, after entering the circulation, activate immune cells and intensify low-grade inflammation [16]. Pro-inflammatory cytokines can cross the blood–brain barrier or interact with it indirectly, activating microglia and triggering a neuroinflammatory response, which is considered one of the key pathomechanisms of delirium. At the same time, reduced production of SCFAs, such as butyrate, weakens the regeneration of the intestinal epithelium and promotes further aggravation of intestinal barrier disorders [17,18,19]. Tryptophan metabolism also plays an important role, which, in conditions of dysbiosis, is shifted towards the kynurenine pathway. The metabolites formed in its course, such as 3-hydroxykynurenine or quinoline acid, have neurotoxic effects and can disrupt the functioning of structures responsible for attention, memory and the regulation of consciousness. In contrast, beneficial metabolites of the microbiota, such as indole-3-propionic acid (IPA), exhibit neuroprotective properties, and their reduced levels—seen in patients with POD—may increase the brain’s susceptibility to oxidative stress and damage [18]. The gut–brain axis also includes neuronal signaling, including vagus nerve activity, which responds to changes in the gut environment and transmits information to limbic structures and the brainstem [20]. Dysbiosis can disrupt this signaling, leading to changes in mood regulation, stress response, and cognitive processes. In addition, the microbiota affects the synthesis and degradation of neurotransmitters such as serotonin, dopamine and GABA, the balance of which is crucial for the proper functioning of the CNS. Disruption of these pathways in the postoperative period may promote disorganization of cognitive processes and increase the risk of delirium [19,21]. In light of the available data, the gut–brain axis is one of the most important areas of research on the pathogenesis of postoperative delirium. Understanding the mechanisms linking the microbiota to brain function opens up new therapeutic possibilities, including microbiota modulation through diet, probiotics, prebiotics, or pharmacological interventions targeting bacterial metabolites [22]. Incorporating these strategies into clinical practice may contribute to reducing the incidence of POD and improving treatment outcomes for patients undergoing surgery in the future [19,21].

### Objectives and Rationale

The aim of this review was to assess the association between the gut microbiota and the occurrence of POD. Particular attention was paid to factors that play a key role in the development of POD from the perspective of the gut microbiota. The research question (RQ) formulated as part of the scoping review is as follows: does the gut microbiota influence the occurrence of POD?

## 2. Materials and Methods

### 2.1. Study Design

We selected the scoping review approach because our aim was to outline and organize the key concepts related to the role of the gut microbiota in POD. Scoping reviews represent a relatively recent methodological framework, and there is still limited guidance on how to choose between a systematic review and a scoping review when synthesizing evidence, particularly in areas where the literature is extensive, complex, heterogeneous, or has not yet been thoroughly examined [23]. Our scoping review was conducted in accordance with the methodology outlined in the Joanna Briggs Institute Manual for Scoping Reviews and followed the recommendations of the Preferred Reporting Items for Systematic Reviews and Meta-Analyses for Scoping Reviews (PRISMA-ScR) guidelines [24,25].

### 2.2. Inclusion and Exclusion Criteria

To precisely define the key elements related to the gut microbiota and postoperative delirium, we formulated a research question that clearly defined the population, the central concept, and the context defining the scope of our review.

The inclusion criteria were as follows: articles published in 2015–2025; original articles (observational and randomized trials), meta-analyses, and systematic and narrative reviews; articles with access to the full text; English-language articles; and human and animal research.

The exclusion criteria included the following: publications older than 10 years; case reports, comments, letters to the editor, and book chapters; no full-text articles; and articles in a language other than English.

One animal study was intentionally included as a supplementary source of mechanistic data.

Population

This review included studies describing the influence of the gut microbiota on the occurrence of POD. In this review, the gut microbiota was defined as the complex, symbiotic community of microorganisms—predominantly bacteria, but also fungi, archaea, and viruses—residing in the human gastrointestinal tract. POD is an acute, fluctuating disturbance in attention, awareness, and cognition developing within days of surgery, often characterized by memory impairment, disorganized thinking, and altered consciousness [4,6,13,16].

In patients undergoing surgery, the occurrence of POD and the composition of the gut microbiota before or after surgery were evaluated.

Concept

The focus was on assessing the influence of the intestinal microbiota on the occurrence of postoperative delirium. The aim of this review was to assess the association between the gut microbiota and the occurrence of postoperative delirium.

The influence of the gut microbiota on the development of POD, including dysbiosis, microbiota metabolites (SCFA, indoles, and TMAO), intestinal barrier integrity, and gut–brain axis was also investigated.

Context

The studies included in this review included patients after surgery.

The clinical context included the perioperative period (preoperative, intraoperative, and postoperative), various types of procedures (orthopedics, cardiac surgery, oncological surgery, and abdominal surgery), and hospital conditions.

Types of studies

This review included a retrospective observational study of any design or methodology.

To ensure methodological consistency,

- Population keywords included: surgery, postoperative, and perioperative;
- Keywords for the concept included: gut microbiota, dysbiosis, SCFA, indole, IPA, and TMAO;
- Context keywords included: POD, delirium, and acute confusional state.

All three components of the PCC had to be present for this study to be included.

### 2.3. Search Strategy

Three authors searched the following databases: PubMed, Scopus, EBSCO, Web of Science, Google Scholar, and the Cochrane Library. The following keywords were used: “gut microbiota”, “delirium”, “postoperative delirium”, “delirium and gut microbiota”, “gut microbiota after surgery”, “delirium after surgery”, “gut-brain axis”, “dysbiosis”, and “short-chain fatty acids”. We used keywords and their combinations with the operators AND and OR. All records were initially screened by title and abstract to remove studies that did not meet the criteria. Any disagreements were resolved through discussion among the four investigators, and by the end of the selection process, full consensus was reached on the articles to be included. The initial search began on 20 December 2025, and the final search was conducted on 10 January 2026. Detailed search strategy used in this review shows Table 1.

### 2.4. Extraction of Data

A data extraction form based on the JBI scoping review guidelines [24] was used to collect key information from the included studies. The extraction process—referred to as “data charting” in the scoping review methodology [26]—was carried out independently by two reviewers. To guide the identification of relevant studies, we applied the Population–Concept–Context (PCC) framework. The extracted data included the first author, publication year, country, study design, study aim, inclusion and exclusion criteria (PCC), outcomes, and main findings. All data were organized using Microsoft Excel.

### 2.5. Critical Appraisal Process

A scoping review can summarize the existing body of evidence without requiring a formal methodological appraisal of the included studies [24].

### 2.6. Process for Including Publications in the Review

**Figure 1:**
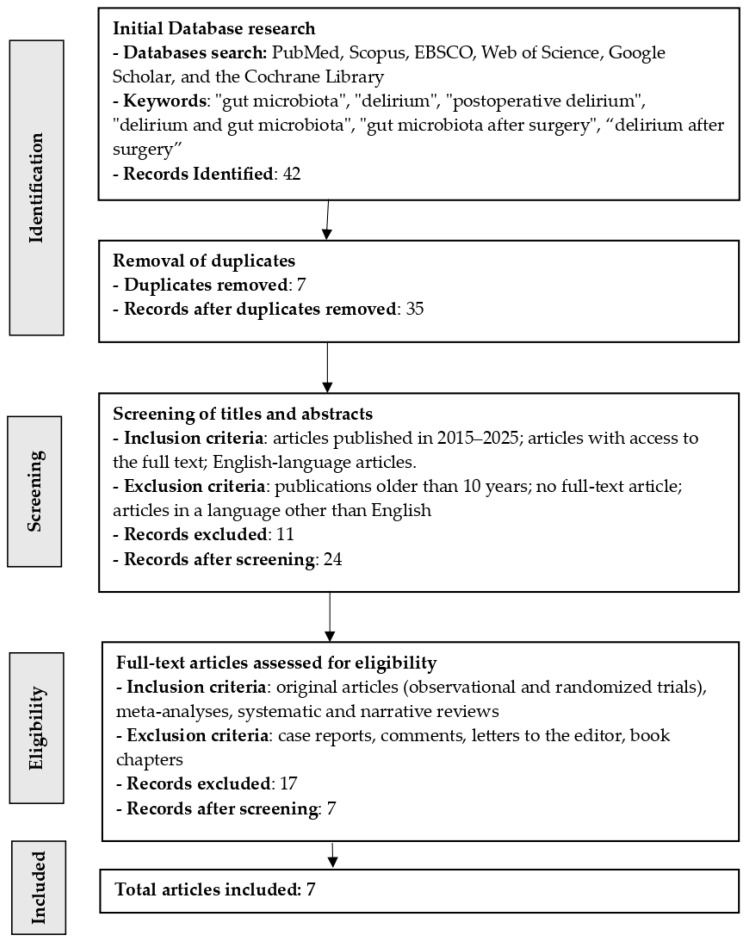
Literature search and selection flowchart for this review.

In our coverage review, we initially identified 42 articles, of which 7 articles on the influence of the gut microbiota on POD were ultimately included (Figure 1). After removing duplicates (*n* = 7), 35 articles remained. After reviewing the articles according to the inclusion and exclusion criteria (*n* = 11), 24 articles remained. Seventeen publications lacked full text and were excluded, leaving 7 papers. As a result, after meeting all requirements, 7 publications were included in this review. This research was conducted in the USA (*n* = 2) and China (*n* = 5). The results are presented in Figure 1 and Table 2.

## 3. Main Tasks of the Intestinal Flora in the Body in the Perioperative Period

The gut flora performs a number of key functions in the human body, which together form the foundation of metabolic, immune and neurological health [34]. Its tasks include, above all, supporting digestion by breaking down ingredients that the body cannot digest on its own, producing short-chain fatty acids that nourish intestinal cells, and synthesizing certain vitamins, such as vitamin K or B vitamins. In addition, it protects against pathogenic microorganisms, competing with them for space and nutrients and producing substances that inhibit their development [35,36]. It also affects metabolism and energy management, affects body weight, and through the gut–brain axis, participates in the regulation of mood, stress response and cognitive functions [37]. Finally, it is involved in neutralizing certain toxins and metabolites, supporting the body’s detoxification processes [38].

### 3.1. Supporting Digestion and Metabolism

Supporting digestion and metabolism by the intestinal flora is one of the most fundamental and, at the same time, the most complex aspects of its functioning. The gut microbiota functions as a highly specialized bioreactor that complements the limited enzymatic capabilities of humans. In practice, this means that intestinal bacteria break down nutrients that the human digestive system cannot digest on its own, primarily various fractions of dietary fiber, plant polysaccharides or resistant starches [39]. The product of this process are short-chain fatty acids such as butyrate, propionate and acetate, which play a key metabolic role: they are the main source of energy for intestinal epithelial cells, modulate glucose and lipid metabolism, affect insulin sensitivity and regulate inflammatory processes. Thanks to this, the microbiota not only supports the local functioning of the intestines but also affects the metabolism of the whole body. An important element of supporting digestion is also the participation of bacteria in the metabolism of proteins and fats [40]. Although excessive bacterial proteolysis can be disadvantageous, a properly balanced microbiota participates in the processing of amino acids and the production of metabolites with signaling action. Similarly, in the case of fats, microorganisms affect their absorption and modulate the secretion of intestinal hormones, such as GLP-1 or PYY, which regulate appetite and metabolic rate [41]. The microbiota also participates in the conversion of bile, which affects the digestion of lipids and the regulation of bacterial composition through the antibacterial effect of bile acids. Supporting metabolism also includes the synthesis of vitamins, especially vitamin K and some B vitamins, which are essential for the proper functioning of the nervous, hematopoietic and energy systems. Moreover, the microbiota influences the bioavailability of many nutrients, including minerals, by modifying the pH of the gut and producing metabolites that facilitate their absorption [39,41]. All these processes make the intestinal flora an integral part of metabolic homeostasis; it plays a role in weight regulation, glucose control, and even in the risk of developing metabolic diseases, such as obesity or type 2 diabetes. As a result, supporting digestion and metabolism by the microbiota is not just an addition to human physiology, but one of the key mechanisms for maintaining health at both the local and systemic levels [42].

### 3.2. Mechanisms of Influence of the Gut Microbiota on the Development of Postoperative Delirium

The gut microbiota plays a key role in the pathophysiology of POD, and the mechanism of its influence includes a complex network of interactions in the gut–brain axis, modulation of the immune response, and regulation of neuroactive metabolites [43,44,45,46]. Microbiota disorders, caused by surgical stress, antibiotic therapy, diet change or immobilization, lead to dysbiosis, characterized by a decrease in bacterial diversity and a decrease in the number of species with anti-inflammatory properties, such as Faecalibacterium prausnitzii or Bifidobacterium [47]. This dysbiosis results in increased permeability of the intestinal barrier, which allows the translocation of lipopolysaccharide (LPS) and other bacterial components into the circulation [48,49,50,51]. The presence of these molecules activates toll-like receptors (TLRs), particularly TLR4, initiating a pro-inflammatory cascade involving cytokines such as IL-6, TNF-α and IL-1β. These cytokines cross the blood–brain barrier or interact with it indirectly, leading to microglial activation and neuroinflammation, which is one of the main pathogenetic mechanisms of delirium. At the same time, intestinal dysbiosis disrupts the production of short-chain fatty acids (SCFAs), especially butyrate, which has a neuroprotective function, strengthens the intestinal barrier and modulates microglial activity [45,46,47,48,49]. SCFA deficiency promotes increased inflammation and increases the brain’s susceptibility to operational stress and hypoxia. The microbiota also influences tryptophan metabolism by shifting it towards the kynurenine pathway, leading to increased production of neurotoxic metabolites such as 3-hydroxykynurenine and quinolic acid [51,52,53,54]. These metabolites disrupt the balance of neurotransmitters, increase excitotoxicity and promote neuronal dysfunction characteristic of delirium. In addition, intestinal bacteria modulate the production of serotonin and GABA, and their impaired synthesis affects the regulation of circadian rhythm, mood and cognitive functions, which are particularly sensitive in the perioperative period [55,56]. It is worth noting that surgery and the associated stress factors can themselves disrupt the microbiota, creating a vicious circle: dysbiosis increases the risk of delirium, and delirium and metabolic stress exacerbate microbial disorders. A growing body of research indicates that the gut microbiota can modulate the neuroendocrine response by influencing the hypothalamic–pituitary–adrenal axis, increasing cortisol levels, and increasing susceptibility to postoperative consciousness disorders [48,57,58]. As a result, the gut microbiota is becoming one of the key regulators of a patient’s susceptibility to POD, and its modulation—through diet, probiotics, prebiotics or interventions to reduce dysbiosis—represents a promising direction in the prevention and therapy of POD, although it requires further clinical trials of high methodological quality [53].

Another important mechanism is the modulation of tryptophan metabolism. Under dysbiosis, the metabolism of this amino acid is shifted towards the kynurenine pathway, which leads to increased production of neurotoxic metabolites such as 3-hydroxykynurenine or quinolic acid [44,54,55,56,57]. These metabolites potentiate excitotoxicity through excessive activation of NMDA receptors, promote oxidative stress, and disrupt neurotransmitter balance, which is an important element of the pathophysiology of delirium. At the same time, the availability of tryptophan for serotonin synthesis decreases, which can affect circadian rhythm, mood and cognitive function disorders, especially in the perioperative period [49,50,51,58]. The gut microbiota also affects the functioning of the hypothalamic–pituitary–adrenal (HPA) axis. Dysbiosis can lead to excessive activation of this axis, increasing the secretion of cortisol, which intensifies inflammation, impairs neuroplasticity and promotes the development of delirium. It has also been shown that the microbiota modulates the production of neuroactive substances such as GABA, dopamine and serotonin, which play a key role in regulating consciousness, attention and behavior [57,58,59]. It is worth noting that surgery itself can induce changes in the microbiota, creating a vicious circle: dysbiosis increases the risk of delirium, and delirium and the associated metabolic stress exacerbate microbial disorders [60,61,62]. As a result, the gut microbiota becomes one of the key factors modulating the patient’s susceptibility to postoperative delirium. Understanding these mechanisms opens up new therapeutic possibilities, including microbiota modulation through dietary interventions, probiotics, prebiotics, synbiotics, and strategies to reduce dysbiosis in the perioperative period [63]. While the results of the studies are promising, further, well-designed clinical trials are needed to conclusively determine the effectiveness of such interventions in preventing and treating POD [64,65].

### 3.3. Perioperative-Specific Microbiota Disorders and the Risk of Postoperative Delirium: The Role of Antibiotic Therapy, Surgical Stress, Fasting, and Neurotransmission Disorders

The perioperative period is one of the most taxing physiological experiences for the human body, involving complex interactions between surgical stress, inflammatory response, metabolic disorders and changes in the gut microbiota [20]. A growing body of evidence indicates that it is the microbiota—its stability, diversity and ability to produce neuroactive metabolites—that plays a key role in modulating the risk of POD. The studies included in this review have repeatedly emphasized that “POD is associated with gut microbiota dysbiosis, marked by an increase in opportunistic pathogens and a decrease in SCFA-producing genera”, indicating the fundamental importance of microbial disorders in the pathogenesis of post-operative disorders of consciousness [20,21].

Antibiotics are an integral part of perioperative prevention and treatment, but their impact on the microbiota is profound, rapid and often long-lasting. Even a single dose of antibiotic can lead to a sharp decrease in microbial diversity, elimination of commensal bacteria and excessive growth of opportunistic pathogens such as Enterococcus, Klebsiella or Clostridioides [64]. One of the studies included in this review showed that patients with POD had significantly increased abundance of Enterococcus, which is consistent with the observation that antibiotics promote the dominance of species with high pro-inflammatory potential. Antibiotic therapy also leads to the reduction of bacteria producing short-chain fatty acids (SCFAs), such as Faecalibacterium, Ruminococcus or Bacteroides [65]. SCFAs—especially butyrate—play a key role in maintaining the integrity of the intestinal barrier, modulating the immune response and regulating microglial activity. Their deficiency promotes increased intestinal permeability (“leaky gut”), which enables the translocation of lipopolysaccharide (LPS) into the circulation. LPS activates TLR4 receptors, initiating a cascade of pro-inflammatory cytokines (IL-6, TNF-α, and IL-1β), which can cross the blood–brain barrier and induce neuroinflammation—one of the main mechanisms leading to delirium [27,28]. It is worth noting that antibiotics also affect the metabolism of tryptophan, shifting it towards the kynurenine pathway, which leads to an increase in the concentration of neurotoxic metabolites such as 3-hydroxykynurenine or quinoline acid. These disorders can exacerbate excitotoxicity and neuronal dysfunction, especially in the structures responsible for attention and consciousness.

Surgical stress includes both physiological (tissue injury, blood loss, and hypoxia) and neuroendocrine (HPA axis activation). An increase in cortisol, adrenaline and norepinephrine directly affects the composition of the microbiota, promoting the growth of pathogenic bacteria and the reduction in commensal species. This stress also leads to changes in intestinal motility, perfusion and mucus secretion, which further destabilizes the microbial environment [28]. Activation of the HPA axis affects the microbiota through immune and metabolic mechanisms. Cortisol increases the permeability of the intestinal barrier, which promotes the translocation of bacterial PAMPs (pathogen-associated molecular patterns) into the bloodstream. As a result, microglia are activated, and neuroinflammation intensifies, which is a key element of the pathogenesis of POD [44]. Surgical stress also affects neurotransmission. Increased glutamatergic activity, decreased cholinergic activity and disorders in the GABAergic system are typical of delirium. The gut microbiota modulates these systems by producing neuroactive metabolites such as GABA, serotonin, dopamine and indoles. One study showed that lower concentrations of indole-3-propionate (IPA)—a metabolite with potent neuroprotective properties—were inversely correlated with the risk of POD. IPA protects neurons from oxidative stress and supports mitochondrial biogenesis by activating PGC-1α, which may explain its protective effects [64].

Preoperative fasting, often lasting 8–12 h, and in practice often longer, leads to significant changes in the microbiota. The lack of nutrient substrates for commensal bacteria results in a decrease in SCFA production and an increase in bacteria using alternative energy sources, including intestinal mucus. This phenomenon can lead to thinning of the mucin layer and increase susceptibility to bacterial translocation. In addition, fasting increases cortisol and catecholamine levels, which—like surgical stress—promotes dysbiosis. Studies in animal models have shown that starvation leads to an increase in pro-inflammatory bacteria, such as Enterobacteriaceae, and a decrease in butyrate-producing bacteria. In the context of POD, it is particularly important that fasting disrupts the metabolism of tryptophan and serotonin. A deficiency of nutrient substrates leads to a decrease in serotonin synthesis in the intestines, which affects the regulation of circadian rhythm, mood and cognitive functions, areas that are particularly sensitive in the postoperative period [27,28,29,30,31].

After surgery, the microbiota undergoes rapid changes. One study in an animal model concluded that “abnormal gut microbiota composition after abdominal surgery may contribute to the development of POD”, confirming that dysbiosis is not just a marker, but a potential causative factor. Postoperative dysbiosis includes a decrease in microbial diversity, an increase in opportunistic pathogens, a decrease in SCFA-producing bacteria, disorders of amino acid metabolism (arginine, histidine, and ornithine), and a shift in tryptophan metabolism towards neurotoxic metabolites. These changes lead to microglial activation, neurotransmission disorders, oxidative stress, and mitochondrial dysfunction, all key components of the pathogenesis of delirium [32,44].

The microbiota affects the nervous system through the production of neuroactive metabolites (GABA, serotonin, and dopamine), modulation of tryptophan metabolism, regulation of vagus nerve activity, influence on the integrity of the blood–brain barrier, and modulation of microglia [28]. In the perioperative period, disorders occur in each of these areas. Dysbiosis leads to a decrease in serotonin (circadian rhythm disturbances and attention), a decrease in GABA (intensification of arousal), an increase in glutamate (excitotoxicity), an increase in neurotoxic kynurenine metabolites, and disorders in dopaminergic regulation (disorganization of thinking). All these changes create an environment conducive to the development of delirium, especially in elderly patients, with comorbidities or reduced cognitive reserve [44,63,64].

Table 3 summarizes the key bacterial taxa and metabolites associated with the occurrence of POD in the studies included in this review. The direction of changes (increased/decreased abundance or concentration), the assigned role (risk factor vs. protective factor), the strength and nature of the association, and the most important mechanistic implications in the context of the gut–brain axis, SCFA, tryptophan and arginine metabolism, and perioperative dysbiosis are compared.

## 4. Main Factors Associated with Gut Microbiome That Influence the Development of Postoperative Delirium

The main factors associated with the gut microbiome that influence the development of POD include complex interactions between gut flora composition, intestinal barrier integrity, metabolism of neuroactive substances, and regulation of the inflammatory response [6]. In the perioperative period, dysbiosis often occurs due to surgical stress, antibiotic therapy, dietary changes or immobilization, which leads to an imbalance between commensal bacteria and potentially pathogenic microorganisms. Such a change in the composition of the microbiota promotes increased production of bacterial toxins, including lipopolysaccharide, which can penetrate the weakened intestinal barrier into the bloodstream, intensifying the systemic inflammatory response. Inflammation, especially of a low-grade nature, is one of the key pathophysiological mechanisms leading to disorders of the central nervous system and the development of delirium [9,11]. An important factor is also the influence of the microbiota on the metabolism of short-chain fatty acids, such as butyrate, which play a role in maintaining the integrity of the intestinal barrier and modulating the immune response. Their deficiency, resulting from dysbiosis, promotes increased intestinal permeability and increased penetration of inflammatory mediators into the circulation. In addition, the microbiota participates in the synthesis and degradation of neuroactive substances such as serotonin, GABA or dopamine, as well as in the metabolism of tryptophan, the disruption of which can lead to increased production of neurotoxic metabolites of the kynurenine pathway. In the postoperative period, when the neurochemical balance is particularly sensitive, such changes can promote disorders of consciousness [14]. Another element is the influence of the microbiota on the functioning of the gut–brain axis, which is responsible for two-way communication between the digestive tract and the central nervous system. Dysbiosis can disrupt nerve and hormonal signaling, leading to changes in the reactivity of the limbic system and the centers that regulate attention and orientation. Finally, the microbiota modulates the immune response, and its disruption can lead to overactivation of the microglia, which promotes neuroinflammation, a process strongly linked to the pathogenesis of delirium [18,22]. As a result, factors related to the gut microbiome are an important element of the risk of developing POD, and their role is increasingly being analyzed as a potential target for preventive and therapeutic measures.

The gut–brain axis influences the development of POD through complex, two-way communication between the gastrointestinal tract and the central nervous system, which is significantly disrupted in the perioperative period [66]. The gut microbiota, which is a key component of this axis, regulates neurochemical, immune, and metabolic processes, and its destabilization—typical after surgery—can initiate mechanisms that lead to impaired consciousness. Dysbiosis caused by surgical stress, antibiotic therapy, changes in diet or immobilization leads to a weakening of the intestinal barrier and increased permeability, which promotes the penetration of bacterial toxins and inflammatory mediators into the bloodstream [67]. These pro-inflammatory signals can activate microglia in the brain, triggering a neuroinflammatory response, which is one of the key pathomechanisms of delirium. At the same time, the microbiota influences the metabolism of neuroactive substances such as serotonin, dopamine or GABA, as well as the tryptophan–kynurenine pathway, the disruption of which can lead to increased production of neurotoxic metabolites [68]. In the postoperative period, when the neurochemical balance is particularly sensitive, such changes can disrupt the functioning of the centers responsible for attention, orientation and regulation of consciousness. The gut–brain axis also includes nerve signaling through the vagus nerve, which responds to changes in the gut environment and transmits them directly to brain structures [69]. Dysbiosis can disrupt this flow of information, leading to abnormal modulation of the stress response and increased susceptibility to cognitive impairment. Finally, the microbiota modulates the immune response, and its destabilization can lead to overactivation of the immune system and chronic inflammation of low severity that favors neuronal dysfunction [66,67]. As a result, disorders of the gut–brain axis in the postoperative period create an environment conducive to the development of delirium through the synergistic action of dysbiosis, neuroinflammation, neurotransmission disorders and abnormal nerve signaling [70]. Further cause-and-effect research should be conducted before further interventions are undertaken.

### Intestinal Bacteria Affecting the Onset of Postoperative Delirium

The development of POD is increasingly being analyzed in the context of disorders of the intestinal microbiota, and available studies indicate that specific groups of bacteria—both those with protective effects and those promoting inflammation—can significantly modulate the risk of impaired consciousness after surgery [71]. There is no single “pathogenic” species responsible for delirium, but rather a characteristic microbial profile in which there is a decrease in anti-inflammatory and neuroprotective bacteria and an increase in pro-inflammatory, endotoxic and metabolism-disrupting microorganisms. The following text expands on these relationships, presenting the most important groups of intestinal bacteria associated with the pathogenesis of POD. One of the best documented microbiotic factors increasing the risk of delirium is the reduction in the number of bacteria producing short-chain fatty acids, especially butyrate [71,72]. This group primarily includes *Faecalibacterium prausnitzii*, *Roseburia* spp. and *Eubacterium rectale*. These bacteria play a key role in maintaining the integrity of the intestinal barrier, modulating the immune response, and regulating microglial function in the central nervous system. Butyrate has an anti-inflammatory effect, stabilizes intercellular connections in the intestinal epithelium and limits the penetration of lipopolysaccharide and other bacterial toxins into the bloodstream [73]. In the perioperative period, when the patient is exposed to surgical stress, starvation, antibiotic therapy and changes in peristalsis, the number of butyrogenic bacteria often decreases rapidly. This results in increased intestinal permeability, increased inflammation and activation of the gut–brain axis in the neuroinflammatory direction, which is one of the key mechanisms leading to delirium [71,72].

The second important group of bacteria is pro-inflammatory microorganisms, especially those belonging to the Enterobacteriaceae family, such as *Escherichia coli*, *Klebsiella pneumoniae* or *Enterobacter cloacae*. In conditions of postoperative dysbiosis, these bacteria tend to multiply excessively, especially in a situation of weakening the colonization resistance of the intestines [74]. Enterobacteriaceae produce large amounts of endotoxins, including lipopolysaccharide, which, when entering the bloodstream, activates the immune system and leads to a systemic inflammatory response. Such inflammation can penetrate the central nervous system, activating microglia and disrupting the functioning of neurons, which promotes the development of disorders of consciousness [73]. The growth of Enterobacteriaceae is especially common in patients undergoing broad-spectrum antibiotic therapy, which destroys commensal bacteria, leaving room for opportunistic microbes.

Another group of bacteria associated with delirium is microorganisms that modulate tryptophan metabolism, especially some species of the genus *Clostridium* and *Bacteroides*. Disruption of tryptophan metabolism can shift its transformations towards the kynurenine pathway, leading to increased production of neurotoxic metabolites such as 3-hydroxyquinurenin or quinolic acid. These substances are known to have neurodegenerative, pro-inflammatory and cognitive impairment effects. In the postoperative period, when the neurochemical balance is particularly sensitive, even small changes in tryptophan metabolism can lead to impaired consciousness, especially in the elderly or with comorbidities [71].

The decline in the number of immunomodulatory and neuroprotective bacteria, such as *Bifidobacterium* and *Lactobacillus*, also plays an important role. These microorganisms support the production of anti-inflammatory cytokines, strengthen the intestinal barrier and participate in the synthesis of neuroactive substances, including serotonin. Their deficiency leads to a weakening of colonization resistance, increased susceptibility to colonization by pathogens and disorders in the gut–brain axis. Studies have observed that patients with low levels of *Bifidobacterium* are more likely to show symptoms of cognitive impairment, and their microbiota has a profile conducive to neuroinflammation [71,72].

It is also worth paying attention to bacteria producing metabolites with toxic or pro-inflammatory effects, such as some strains of *Bacteroides fragilis* in pathogenic forms or species of the genus *Ruminococcus* associated with the degradation of intestinal mucus [73]. Excessive activity of these bacteria can lead to weakening of the intestinal mucosa, increased exposure of the epithelium to toxins and microorganisms, and increased inflammation. As a result, the intestinal barrier is disrupted, which promotes bacterial translocation and activation of the immune system [74].

The development of POD is closely related to characteristic changes in the intestinal microbiota. The most important are a decrease in SCFA-producing bacteria, an increase in pro-inflammatory bacteria from the Enterobacteriaceae family, disorders of tryptophan metabolism associated with some species of *Clostridium* and *Bacteroides*, and a deficiency of neuroprotective bacteria such as *Bifidobacterium* and *Lactobacillus* [75]. It is not a single pathogen, but the overall shift of the microbiome towards a pro-inflammatory and neurotoxic profile that creates an environment conducive to the onset of delirium after surgery. With the development of research on the microbiota, it is becoming increasingly clear that modulation of the composition of the intestinal flora may become one of the key elements of prevention and therapy of consciousness disorders in the perioperative period in the future [76]. Table 4 shows major bacteria involved in the construction of the gut microbiome and influencing POD, with microscopic images of the main groups.

## 5. Limitations and Future Research

This scoping review has several important limitations that need to be taken into account when interpreting its results. First, the number of studies directly examining the relationship between the gut microbiota and the prevalence of POD is still limited, and most of the included publications come from two countries: China and the United States. Such geographic concentration may affect the limited ability to generalize the results to populations with different eating habits, health systems or genetic conditions. In addition, the significant heterogeneity of research designs, types of surgery, patient populations and methods for assessing the microbiota makes it difficult to make direct comparisons and synthesize the results. Differences in sampling time (preoperative vs. postoperative), sequencing techniques and taxonomic levels of analysis can affect the results obtained and limit their reproducibility. Most of the studies included are observational, which makes it impossible to unambiguously establish a cause-and-effect relationship. Although correlations have been observed between specific bacterial taxa and the occurrence of delirium, the mechanisms underlying these relationships remain largely hypothetical and require further experimental investigation. Finally, perioperative factors such as antibiotic use, type of anesthesia, nutritional status and bowel preparation were not consistently analyzed in all studies, even though they can significantly affect the composition of the microbiota and the risk of delirium. Their role as potential modifiers should be taken into account in future research projects. Despite these limitations, this review points to a growing body of evidence supporting the involvement of the gut microbiota in the pathogenesis of POD and highlights the need for standardized, multicenter studies to better understand this relationship and develop microbiota-targeted therapeutic strategies.

### Limitations of Extrapolating Results from Animal Models

Animal models, such as the study by Zhang J. et al. (2019) [33], provide valuable information on the biological mechanisms underlying the relationship between the gut microbiota and the development of postoperative delirium. However, their interpretation in the context of the human population comes with significant limitations, which should be clearly emphasized.

*Species differences in the composition and functioning of the microbiota*

The microbiota of mice differs significantly from the human microbiota in terms of species composition, proportions of dominant bacteria, bacterial metabolism, and responses to diet and stress. This means that the mechanisms observed in animals may not fully reflect the processes that occur in humans.

*A different neuroimmune response*

The immune and nervous systems of rodents respond differently to inflammatory and stress stimuli than in humans. In the context of delirium, this is particularly important because the activation of microglia, the permeability of the blood–brain barrier, and the response to cytokines can be different, which limits the possibility of direct transmission of results.

*Differences in the course of surgical procedures and surgical stress*

Animal models use simplified surgical procedures that do not reflect the complexity of surgeries performed in humans, the variety of anesthesia factors, the disease burden of patients, and the influence of age, multimorbidity and medications. This makes the animal’s body’s response to the procedure less complex than that of a clinical patient.

*Limited ability to assess delirium*

In animals, delirium cannot be assessed in the same way as in humans. Only indirect behavioral indicators are used, which do not fully reflect the complexity of consciousness disorders; they can only be interpreted as analogous models, and not equivalent to clinical delirium.

*The function of animal studies is complementary, not conclusive*

Studies on animal models are extremely valuable because they allow us to study pathophysiological mechanisms, enable the control of variables impossible in clinical trials, and are the starting point for the formulation of hypotheses. However, their results cannot be treated as clinical evidence, but only as support for the interpretation of studies in humans.

## 6. Implications for Generalizability

Most of the available studies come from two countries, China and the United States, which limits the possibility of transferring the results to populations with different dietary patterns, lifestyles, environmental exposures and microbiota structures. As one study pointed out, “POD is associated with gut microbiota dysbiosis, marked by an increase in opportunistic pathogens and a decrease in SCFA-producing genera”; however, microbiota composition and dominant taxa differ significantly between geographic populations, which may affect the direction and strength of the observed relationships.

The variety of surgical procedures (orthopedics, cardiac surgery, oncological surgery, and abdominal procedures) and the diversity of perioperative protocols (antibiotic therapy, bowel preparation, fasting time, and type of anesthesia) make it difficult to clearly determine which elements of dysbiosis are universal and which are specific to a given type of procedure. For example, one study showed that “mechanical bowel preparation increased the incidence of POD”, suggesting that some interventions may have unique effects on the microbiota and the risk of POD, difficult to generalize to other clinical contexts.

Most of the studies included older people, often with multiple morbidities, which limits the possibility of transferring the results to younger adults or patients without burdens. Aging is one of the main factors modulating the microbiota and also increasing susceptibility to cognitive impairment. Therefore, it is unclear whether the observed relationships are universal or specific to the geriatric population.

Differences in microbiota analysis methodology (different sequencing platforms, different bioinformatics approaches, and a lack of standardization in sampling) may affect the comparability of results. Some studies analyzed the preoperative microbiota, others analyzed the postoperative microbiota, and others analyzed circulating metabolites, which further complicates the synthetic generalization of the data.

The nature of observational research makes it impossible to unambiguously determine the causal relationship. Although an animal model has shown that “abnormal gut microbiota composition after abdominal surgery may contribute to the development of POD”, interventional studies are still lacking in the human population to confirm that microbiota modulation can reduce the risk of delirium. Thus, generalizing the results to clinical practice requires caution.

The diversity of taxa and metabolites associated with POD indicates that there is no one-size-fits-all “microbiotic risk phenotype.” In some studies, opportunistic bacteria (Enterococcus, Bacteroides, and Veillonella) played a key role, in others, the decline in SCFA producers did, and in others, metabolites such as indole-3-propionic acid (IPA) or arginine did. This heterogeneity suggests that the mechanisms leading to POD may be multifactorial and dependent on the clinical context.

The lack of standardization in the assessment of delirium (different scales, different moments of assessment, and different clinical definitions) may affect the variability of outcomes and limit their comparability. Since delirium is a dynamic and fluctuating state, differences in assessment time can lead to an underestimation or overestimation of its frequency. Although the available data indicate a significant association between the gut microbiota and the risk of POD, the generalizability of these results is limited by the heterogeneity of populations, procedures, methodologies and environmental factors. To increase the generalization of results, multi-center studies involving different geographic populations, standardization of methods for microbiota analysis and delirium assessment, interventional studies testing the effectiveness of microbiota modulation, and analyses that take into account confounding factors such as diet, antibiotic therapy, comorbidities, and age are necessary. Only such an approach will allow us to determine which elements of dysbiosis are universal and which are specific to specific groups of patients, which will enable the real use of knowledge about the microbiota in the prevention and treatment of POD.

## 7. Conclusions

This scoping review indicates that the gut microbiota plays an important role in the pathogenesis of POD, and its disorders may be one of the key risk factors for this complication. An analysis of the available studies revealed that dysbiosis—particularly characterized by a decrease in short-chain fatty acid-producing bacteria and an increase in opportunistic microorganisms—promotes increased inflammation, increased intestinal barrier permeability and disorders in the gut–brain axis. These mechanisms can lead to neuroinflammation, dysregulation of neurotransmission, and metabolic disorders, which are closely related to the development of delirium after surgery. The results of the included studies also indicate that specific bacterial taxa—such as Parabacteroides distasonis, Enterococcus, Bacteroides, Romboutsia or Blautia—may be potential markers of delirium risk, while the presence of anti-inflammatory bacteria, such as Olsenella or SCFA producers, may have a protective function. At the same time, a growing body of data suggests that microbiota metabolites, including indoles, arginine and short-chain fatty acids, may be an important link between gut health and central nervous system function in the perioperative period. Despite the growing body of evidence, the current state of knowledge remains fragmented, and the available research is characterized by significant methodological heterogeneity. Furthermore, well-designed prospective studies are needed, including standardized methods for assessing the microbiota, metabolite analysis, and control of perioperative factors such as antibiotic therapy, type of anesthesia and nutritional status. A better understanding of the interaction between the microbiota and the nervous system could enable the development of new preventive and therapeutic strategies in the future, including dietary, probiotic or metabolite interventions, aimed at reducing the risk of POD. The gut microbiota is a promising area of research in the context of POD, and its modulation may become an important component of perioperative care in the future, especially in patients at higher risk. In Table 5, we show a summary of bacteria and POD risks.

## Figures and Tables

**Table 1:** Detailed search strategy used in this review.

Database/Source	Full Search String (Boolean Search String)	Limits/Filters	Notes on PCC
PubMed	“gut microbiota”[MeSH] OR “intestinal microbiota” OR dysbiosis AND “postoperative delirium”[MeSH] OR “postoperative delirium” OR delirium OR “acute confusional state” AND surgery OR postoperative OR perioperative	Years: 2015–2025; Language: English; Full text	P: Patients after surgery C: Gut microbiota and dysbiosis C: Perioperative context
Scopus	TITLE-ABS-KEY “gut microbiota” OR “intestinal microbiota” OR dysbiosis AND TITLE-ABS-KEY “postoperative delirium” OR delirium AND TITLE-ABS-KEY surgery OR postoperative OR perioperative	Years: 2015–2025; Language: English	As above, full compliance with PCC
Web of Science Core Collection	TS = “gut microbiota” OR “intestinal microbiota” OR dysbiosis AND TS = “postoperative delirium” OR delirium AND TS = surgery OR postoperative OR perioperative	Years: 2015–2025; Language: English	As above, full compliance with PCC
Cochrane Library	“gut microbiota” OR “intestinal microbiota” OR dysbiosis AND “postoperative delirium” OR delirium	Years: 2015–2025; Language: English	As above, full compliance with PCC
Google Scholar	“gut microbiota” “postoperative delirium” surgery	Years: 2015–2025; Language: English	Search for grey literature according to PCC

**Table 2:** Characteristics and findings of studies included in this review.

Author, Year	Country	Participants	Findings
Zhang Y. et al., 2023 [27] *	USA	Patients who have had a knee replacement, hip replacement, or laminectomy	✓ Postoperative gut bacteria Parabacteroides distasonis was associated with POD after adjusting for age and sex ✓ The association between delirium and both Prevotella and Collinsella did not meet statistical significance ✓ There may be an association between postoperative gut microbiota, specifically Parabacteroides distasonis, and POD ✓ Further research is needed to better understand the gut–brain axis’s role in postoperative outcomes
Zhou X. et al., 2023 [28] *	USA	Patients who have had a knee replacement, hip replacement, or laminectomy	✓ Indole-3-propionic acid (IPA) was inversely correlated with POD development in humans ✓ Harnessing the gut microbiota and its metabolites could facilitate developing preventive and therapeutic strategies against POD ✓ PGC-1α, a master regulator of mitochondria biogenesis, was implicated in IPA’s role in POD
Huo J. et al., 2025 [29]	China	Elderly patients underwent elective orthopedic surgery	✓ In this study, it was found that the abundance of Romboutsia, Bacteroides faecalis, Blautia mucilaginosa, and Eggerhella lenta in the preoperative microbiota, along with postoperative plasma levels of PA, His, Asp, and Orn, were associated with the risk of POD in elderly patients undergoing orthopedic surgeries ✓ Specific gut microbiota expression may play a potential role in the pathogenesis of POD ✓ Targeting gut microbiota and metabolites may provide new therapeutic strategies for preventing or treating POD
Cao X. et al., 2026 [30]	China	Patients aged ≥65 years who were scheduled for elective lower limb orthopedic surgery	✓ The POD group showed significantly altered gut microbiota ✓ Correlation analysis demonstrated that arginine levels were positively correlated with Megasphaera abundance and CAM-CR score, but negatively correlated with Paraprevotella and Akkermansia abundance ✓ Gut microbiota and arginine–agmatine metabolism are associated with the pathogenesis of POD in elderly lower-limb fracture patients
Huang P. et al., 2025 [31]	China	Patients undergoing off-pump coronary artery bypass grafting	✓ POD is associated with gut microbiota dysbiosis, marked by an increase in opportunistic pathogens and a decrease in SCFA-producing genera ✓ After surgery, POD patients had lower alpha diversity and distinct microbiota compared with non-POD patients ✓ POD was linked to increased opportunistic pathogens (Enterococcus) and decreased SCFAs producers (Bacteroides, Ruminococcus, etc.)
Yang Z. et al., 2022 [32]	China	Patients with gastric cancer	✓ Mechanical bowel preparation increased the incidence of POD ✓ Bacteroides and Veillonella genus might be a risk factor of POD ✓ Genus Olsenella might be a beneficial bacteria to reduce the incidence of POD
Zhang J. et al., 2019 [33] **	China	Mice after abdominal surgery	✓ Abnormal gut microbiota composition after abdominal surgery may contribute to the development of POD ✓ A therapeutic strategy that targets gut microbiota could provide a novel alterative for POD treatment

* Same cohort, different analyses. ** The work was included because of its importance for understanding the pathophysiology, even though it does not meet the human population criterion.

**Table 3:** Summary of key bacterial taxa and metabolites associated with the occurrence of POD in the studies included in this review.

Author (Year)	Taxon/Metabolite	Direction of Change in Patients with POD	Role (Risk/Protection)	Strength/Type of Connection	Mechanistic/Clinical Considerations
Zhang Y. et al., 2023 [27]	Parabacteroides distasonis	Increased abundance postoperatively in patients with POD	Risk factor	Significant relationship after age and gender correction	Suggests the involvement of specific taxa in the gut–brain axis and postoperative outcomes
	Prevotella and Collinsella	Statistically insignificant changes	Ambiguous	No materiality after adjustments	Modulating role possible, requiring larger trials
Zhou X. et al., 2023 [28]	Indol-3-propionowy (IPA)	Lower concentrations in patients with POD	Protective	Inverse correlation with POD development	A metabolite of the microbiota with neuroprotective properties; associated with PGC-1α and mitochondrial biogenesis
Huo J. et al., 2025 [29]	Romboutsia, Bacteroides faecalis, Blautia mucilaginosa, and Eggerthella lenta	Altered abundance preoperatively in patients with POD	Mainly risk factors	Significant associations with POD risk	They indicate the importance of the microbiota profile before surgery in the elderly
	PA, His, Asp, and Orn (plasma metabolites)	Altered postoperative levels in patients with POD	Risk markers	Correlations with the occurrence of POD	They link the microbiota profile to circulating amino acid metabolites
Cao X. et al., 2026 [30]	Megasphaera	Increased abundance, positively correlated with arginine and CAM-CR	Potential risk factor	Significant positive correlation	Indicates the association of arginine–agmatine metabolism with the pathogenesis of POD
	Paraprevotella and Akkermansia	Reduced abundance in patients with POD	Potentially protective	Negative correlation with arginine	Loss of taxa associated with gut barrier integrity and metabolic homeostasis
Huang P. et al., 2025 [31]	Enterococcus	Increased abundance after surgery in patients with POD	Risk factor (opportunistic pathogen)	Significantly higher share in the POD group	Element of dysbiosis with a predominance of opportunistic pathogens after heart surgery
	Bacteroides and Ruminococcus and other SCFA producers	Reduced abundance in patients with POD	Protective	Significantly lower alpha-diversity and the proportion of these genera	Decrease in SCFA production, weakening of the intestinal barrier and increased inflammation
Yang Z. et al., 2022 [32]	Bacteroides and Veillonella	Increased abundance after bowel preparation	Risk factors	Associated with a higher frequency of POD	Mechanical preparation of the intestine increases the frequency of POD; these types may promote pro-inflammatory dysbiosis.
	Olsenella	Reduced abundance in patients with POD	Protective	Inverse association with POD frequency	Potential beneficial taxon reducing the risk of POD
Zhang J. et al., 2019 [33]	Changes in the composition of the postoperative microbiota (mouse model)	Dysbiosis after surgery	Risk factor	Experimental model indicating causation	“Abnormal gut microbiota composition after abdominal surgery may contribute to the development of POD”
	Non-specific taxa/microbiota modulation	Normalization of the microbiota	Protective	Therapeutic effect in the animal model	“A therapeutic strategy that targets gut microbiota could provide a novel alternative for POD treatment”

**Table 4:** Major bacteria involved in the construction of the gut microbiome and influencing postoperative delirium.

Main Group (Type)	Examples of Types	Microscopic Images of the Main Group	Main Tasks	Literature Reference
Firmicutes	*Lactobacillus ^a^*, *Clostridium ^b^*, *Faecalibacterium ^c^*, *Ruminococcus ^d^*	*a* 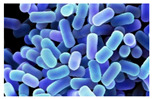 *b* 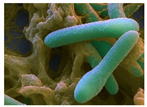 *c* 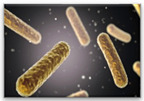 *d* 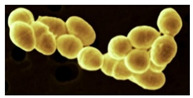	Fiber breakdown, production of short-chain fatty acids (SCFAs), and supporting immunity	[31,71,73]
Bacteroidetes	*Bacteroides ^e^* and *Prevotella ^f^*	*e 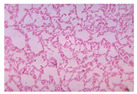 f 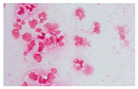 *	Digestion of complex carbohydrates and regulation of metabolism	[29,32,72]
Actinobacteria	*Bifidobacterium ^g^*	*g* 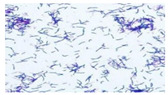	Very important in infants; vitamin production; and protection against pathogens	[27,28,71,73]
Proteobacteria	*Escherichia ^h^* (np. *E. coli*–mainly non-pathogenic strains) and *Klebsiella ^i^*	*h 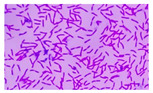 i 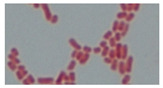 *	Participation in metabolism, but their excess may indicate dysbiosis	[30,32,75,76]
Verrucomicrobia	*Akkermansia muciniphila ^j^*	*j* 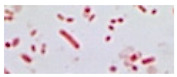	Strengthens the intestinal barrier, and supports glucose and lipid metabolism	[33,74]

The letters in the superscript above the name of the bacterium correspond to the letters from the microscopic image of the given bacterium.

**Table 5:** Summary of bacteria and POD risks.

Gut Microbiota and POD Risk	
***Protective bacteria***: Olsenella Akkermansia Paraprevotella SCFA producers Ruminococcus Bacteroides (SCFA producers) Indole-3-propionic acid (IPA)	***Bacteria associated with POD risk***: Parabacteroides distasonis Enterococcus Veillonella Bacteroides (MBP context) Romboutsia Bacteroides faecalis Blautia mucilaginosa Eggerhella lenta Megasphaera (arginine-linked)
***Protective mechanisms***: SCFA → intestinal barrier IPA → neuroprotection Lower-axle activation HPA	***Risk mechanisms***: Dysbiosis → ↑ LPS → neuroinflammation ↑ pathogensinopportunistic Tryptophan metabolism disorders

## Data Availability

No new data were created or analyzed in this study. Data sharing is not applicable to this article.

## Abbreviations

The following abbreviations are used in this manuscript:

POD	Postoperative delirium
SCFA	Short-chain fatty acid
IPA	Indole-3-propionic acid
TMAO	Trimethylamine N-oxide
CNS	Central nervous system
LPS	Lipopolysaccharide
RQs	Research questions
PRISMA-ScR	Preferred Reporting Items for Systematic Reviews and Meta-Analyses for Scoping Reviews
PCC	Population–Concept–Context
PGC	1α peroxisome proliferator-activated receptor-γ coactivator
CR-CAM	Contrast-ranking class activation mapping algorithm
SCFA	Saccharolytic fermentation of carbohydrates
